# Ethylene Propylene Diene Monomer-Based Composites Resistant to the Corrosive Action of Acetic Acid

**DOI:** 10.3390/ma18194557

**Published:** 2025-09-30

**Authors:** Elena Manaila, Ion Bogdan Lungu, Marius Dumitru, Maria Mihaela Manea, Gabriela Craciun

**Affiliations:** 1 Electron Accelerators Laboratory, National Institute for Laser, Plasma and Radiation Physics, 409 Atomistilor St., 077125 Măgurele, Romania; elena.manaila@inflpr.ro (E.M.); marius.dumitru@inflpr.ro (M.D.); 2 Horia Hulubei National Institute for R&D in Physics and Nuclear Engineering, Multipurpose Irradiation Facility Center—IRASM, 30 Reactorului St., 077125 Măgurele, Romania; ion.lungu@nipne.ro (I.B.L.); mmanea@nipne.ro (M.M.M.)

**Keywords:** EPDM composites, wood sawdust filler, acetic acid resistance, rubber-filler interaction

## Abstract

The potential of elastomeric composites reinforced with natural fillers to replace traditional synthetic materials in applications involving exposure to acidic environments offers both economic and environmental advantages. On the one hand, these materials contribute to cost reduction and the valorization of organic waste through the development of value-added products. On the other hand, the presence of wood waste in the composite structure enhances biodegradation potential, making these materials less polluting and more consistent with the principles of the circular economy. The present study aims to evaluate the behavior of composites based on Ethylene Propylene Diene Monomer (EPDM) synthetic rubber, reinforced with silica and wood sawdust, in a weakly acidic yet strongly corrosive environment—specifically, acetic acid solutions with concentrations ranging from 10% to 30%. The study also investigates the extent to which varying the proportions of the two fillers affects the resistance of these materials under such environmental conditions. Physico-chemical, structural, and morphological analyses revealed that the materials underwent chemical modifications, such as acetylation of hydroxyl groups. This process reduced the hydrophilic character of the sawdust and, combined with the formation of stable interfaces between the elastomeric matrix and the fillers during vulcanization, limited acid penetration into the composite structure. The composites in which 20 phr or 30 phr of wood sawdust were used-replacing equivalent amounts of silica from the initial 50 phr formulation-demonstrated the highest resistance to the corrosive environments. After 14 days of exposure to a 20% acetic acid solution, the composite containing 30% wood sawdust exhibited a decrease in cross-link density of only 1.44%, accompanied by a reduction in Young’s modulus of just 0.95%. At the same time, tensile strength and specific elongation increased by 22.57% and 26.02%, respectively. FTIR and SEM analysis confirmed good rubber-filler interactions and the stability of the composite structure under acidic conditions.

## 1. Introduction

Versatile materials with tunable physical and chemical properties, which can be tailored to specific applications and manufactured at low cost, have attracted significant attention in recent decades [1,2,3,4]. Rubber-based systems-such as rubber–rubber blends, rubber–polymer blends, and composites reinforced with “non-black” fillers (e.g., silica, kaolin, talc, titanium dioxide, or natural fibers)-are known as hybrid composites. These materials offer enhanced mechanical, thermal, electrical, and chemical properties, along with improved durability under various environmental conditions [5,6,7].

Among synthetic rubbers, Ethylene Propylene Diene Monomer (EPDM) stands out for its excellent resistance to heat, oxidation, ozone, and weathering, as well as its elasticity at low temperatures [1,8,9,10,11,12]. Its performance is typically improved via sulfur-based vulcanization, which creates a cross-linked structure [1,13]. EPDM also has a high filler acceptance capacity, allowing for cost-effective reinforcement strategies [1,14,15,16]. The effectiveness of filler dispersion depends on several factors: surface chemistry (functional groups), filler–matrix interfacial interactions (chemical or physical), and the inherent compatibility with the rubber matrix [1,17].

Silica is one of the most commonly used fillers after carbon black in the rubber industry. Its abundance, non-toxicity, thermal stability, and tunable surface chemistry make it highly valuable for reinforcement applications [18,19,20]. Hydrated amorphous silicas, produced via acid precipitation of sodium silicate, are commonly used in the tire industry [18,21,22,23,24]. However, the polar nature of silica leads to strong filler-filler interactions and hydrogen bonding, which result in large, poorly dispersed aggregates in non-polar rubbers like EPDM [18,25,26,27]. Surface modification is often required to improve dispersion and compatibility.

In recent years, natural fillers (bio-fillers) such as hemp, flax, starch, and wood sawdust have gained interest as sustainable alternatives to conventional mineral fillers [28,29,30,31,32,33,34,35,36,37,38,39]. Wood sawdust, in particular, offers benefits such as low cost, biodegradability, favorable processing, and reduced health risks [36,40,41,42]. However, its use also presents challenges. The poor interfacial adhesion between the hydrophilic lignocellulosic filler and the hydrophobic rubber matrix can limit mechanical performance, especially in conventional cross-linking systems [13,32,35,36,37]. The mechanical properties of sawdust-reinforced composites vary significantly with particle size, filler loading, and cross-linking method. Generally, smaller particles enhance mechanical strength by increasing surface area and improving stress transfer at the rubber-filler interface [36,43,44,45,46,47].

This study investigates the behavior of EPDM-based composites reinforced with varying ratios of silica and wood sawdust (50:0 to 0:50 phr) when exposed to acidic environments. The goal was to identify the formulations offering the highest resistance to chemical degradation. Acetic acid was chosen for its dual effect: it is both corrosive and capable of reducing the hygroscopicity of wood sawdust, potentially mimicking the acetylation effect without prior treatment. Although considered a weak acid, its corrosiveness makes it suitable for assessing long-term material stability and potential end-of-life degradation.

The relevance of this work lies in the potential to replace traditional synthetic materials with sustainable elastomeric composites in acidic applications. These systems offer cost reduction, waste valorization, and support for the circular economy. Several novel aspects are introduced: (i) the use of untreated sawdust in acidic media is largely unexplored; (ii) the systematic replacement of silica with sawdust across a full composition range allows for detailed analysis of filler synergy; (iii) FTIR, SEM, and TG/DSC results are correlated with mechanical performance; and (iv) preliminary evidence suggests in situ acetylation effects due to acid exposure.

## 2. Materials and Methods

### 2.1. Materials

The blends used in this study were custom-prepared by Artego S.A., Targu Jiu, Romania, a company specialized in the production and marketing of rubber products. The proportions of the base components-EPDM, silica, and wood sawdust-were selected to comply with the company’s production standard, which specifies 100 phr EPDM and a maximum of 50 phr filler (typically silica).

To investigate the effect of wood sawdust on the composite’s behavior in acidic media, a series of technical rubber sheets were produced via classical vulcanization using benzoyl peroxide. In these formulations, the total filler content remained constant at 50 phr, while silica was partially replaced with wood sawdust.

Six EPDM-based composites with varying filler ratios were prepared using conventional two-roll mixing techniques. The fillers were added gradually into the rubber matrix along with curing agents and other compounding ingredients. The formulations were as follows: EPDM 0: 50 phr silica/0 phr wood sawdust, EPDM 10: 40 phr silica/10 phr wood sawdust, EPDM 20: 30 phr silica/20 phr wood sawdust, EPDM 30: 20 phr silica/30 phr wood sawdust, EPDM 40: 10 phr silica/40 phr wood sawdust, EPDM 50: 0 phr silica/50 phr wood sawdust.

The formulations used to prepare the EPDM-based composites containing silica and/or wood sawdust, as requested by Artego S.A., are presented in Table 1.

From the 300 × 300 mm rubber sheets prepared as described above, using a hydraulic press under controlled temperature and pressure and provided by Artego S.A., dumbbell-shaped specimens were cut for exposure to acetic acid. All analyses and laboratory tests were conducted both before and after the acid treatment.

### 2.2. Laboratory Tests and Analysis

In separate containers for each of the six composite types (EPDM 0 to EPDM 50), eight dumbbell-shaped samples of known mass were immersed in acetic acid solutions with concentrations of 10%, 20%, and 30%, for periods of 3, 7, and 14 days, at a constant room temperature of 25 °C. Of the eight samples in each container, five were used to evaluate swelling, mass loss, and mechanical properties, while the remaining three were used to determine cross-link density in toluene.

#### 2.2.1. Behavior of Swelling and Mass Changes

Swelling and mass variation in acetic acid solutions were evaluated on five dumbbell-shaped samples (type 4) with the following dimensions: total length 35 mm, narrow section length 12 mm, thickness 2 mm, and width 2 mm, at room temperature (23 ± 2 °C) in accordance with ISO 175/2010 [48]. Samples with known initial mass were removed from the acetic acid solutions after the designated exposure times, weighed in their wet state, then dried at room temperature for 24 h, followed by an additional 24 h in a laboratory oven at 80 °C. After drying, the final mass was recorded. The change in composite mass due to immersion in the acidic solution was calculated according to Equation (1) [33,34,49]:

(1) $$Acidic\;solution\;absorption=\;\frac{m_{f}-m_{i}}{m_{i}}\times 100\;$$

where *m_i_* is the initial dry mass of the composite before immersion in the acetic acid solution, and *m_f_* is the mass of the same sample in wet form, measured immediately after extraction from the solution [33,34,49].

#### 2.2.2. Behavior of Cross-Link Density

Cross-link density was evaluated in triplicate by immersing dumbbell-shaped samples (type 4, with dimensions specified in Section 2.1) in toluene, at room temperature (23 ± 2 °C) in accordance with ASTM D6814-02 [50]. The determination was based on the modified Flory-Rehner equation for tetrafunctional networks. Samples of known initial dry mass (*mᵢ*) were immersed in toluene for 72 h, then removed, gently wiped with filter paper to eliminate surface solvent, and weighed in the swollen state (*m_g_*). To minimize solvent evaporation during weighing, the samples were placed in glass vials with ground-glass stoppers. After weighing, the samples were dried at room temperature (25 °C) for 24 h, followed by drying in a laboratory oven at 80 °C for 48 h. After cooling, they were reweighed to determine the final dry mass (*m_s_*). The cross-link density (*ν*) was calculated using the following equation [51,52]:

(2) $$\nu =\frac{Ln(1-\nu_{2m})+\nu_{2m}+\chi_{12}\nu_{2m}^{2}}{V_{1}\left(\nu_{2m}^{1/3}-\frac{\nu_{2m}}{2}\right)}$$

In Equation (2), *V*_1_ is the molar volume of the solvent (106.5 cm^3^/mol for toluene), *ν*_2*m*_ is the volume fraction of polymer in the sample at equilibrium swelling, and *χ*_12_ is the Flory-Huggins polymer-solvent interaction parameter, which for the EPDM-toluene system is 0.393 [51,52].

The volume fraction at equilibrium swelling (*ν*_2*m*_) was calculated from the swelling ratio (*G*) using the following relation:

(3) $$\nu_{2m}=\frac{1}{1+G}$$

(4) $$G=\frac{m_{g}-m_{s}}{m_{s}}\times \frac{\rho_{e}}{\rho_{s}}$$

In Equation (4), *ρ_e_* and *ρ_s_* represent the densities of the composite and the solvent, respectively. The density of toluene (*ρ_s_*) is 0.866 g/cm^3^.

#### 2.2.3. Behavior of Mechanical Properties

Young’s modulus (*E*, MPa), tensile strength (*σ*, MPa), and specific elongation (*ε*, %) were determined according to DIN EN ISO 37:2017 [53] using a Zwick-Roell ProLine Z005 testing machine (Ulm, Germany). Tests were conducted on type 4 dumbbell-shaped specimens (as described in Section 2.1) at a crosshead speed of 200 mm/min. Each measurement was repeated at least five times, and the reported values represent the averages.

#### 2.2.4. Morphological Investigations by Scanning Electron Microscopy (SEM)

The sample surfaces, before and after acetic acid treatment, were examined using Scanning Electron Microscopy (SEM) with a FEI/Phillips microscope (Hillsboro, OR, USA). Fractured samples, prepared by liquid nitrogen cooling and sputter-coated with gold-palladium, were mounted on aluminum stubs and imaged at an accelerating voltage of 30 kV.

#### 2.2.5. Structural Investigations by Fourier Transform Infrared Spectroscopy (FTIR)

The composite structures were characterized by Fourier Transform Infrared (FTIR) spectroscopy using a Bruker Vertex 70 spectrometer (Bruker Optics GmbH & Co. KG, Ettlingen, Germany) equipped with an attenuated total reflection (ATR) Platinum accessory for non-destructive analysis. Measurements were conducted in the spectral range of 4500–450 cm^−1^, with 128 scans averaged and a resolution of 4 cm^−1^.

#### 2.2.6. Thermogravimetric and Differential Scanning Calorimetry (TG/DSC) Analysis

Thermograms of the composites were recorded using the STA 409 PC Luxx simultaneous TG/DSC system (Netzsch-Gerätebau, Selb, Germany). Measurements were performed under nitrogen flow (100 mL/min) using aluminum crucibles (25 µL, max 10 mg). Calibration and analysis followed SR EN ISO 11358:2022 [54] and SR EN ISO 11357-1:2023 [55] standards. Samples were heated from 20 to 590 °C at 10 K/min.

#### 2.2.7. Statistical Analysis

Experimental data are presented as mean ± standard deviation (SD) from three independent measurements. Statistical analysis was conducted using one-way ANOVA followed by Fisher’s Least Significant Difference (LSD) post hoc test. Differences were considered significant at *p* < 0.05. All analyses were performed using Microsoft Excel 2021 and OriginPro V2023.

## 3. Results and Discussion

Before immersion in acetic acid solutions (10%, 20%, and 30%), the composite materials were characterized for swelling, mass change, mechanical properties, structure, and morphology. The results, presented in Table 2, serve as control values for subsequent comparisons.

The composites with partial wood sawdust substitution (EPDM 20 and EPDM 30) showed the highest cross-link density values (3.69 and 2.89 × 10^−4^ mol/cm^3^ respectively) and the lowest solvent uptake (0.446 and 0.391 mL/g), indicating enhanced filler-elastomer interactions and resistance to swelling. Young’s modulus increased with higher wood sawdust content, reaching a maximum of 9.12 ± 1.47 MPa for EPDM 50, which contains only wood sawdust. Tensile strength, however, decreased as the silica content decreased, dropping from 6.25 ± 0.19 MPa (EPDM 0) to 1.02 ± 0.05 MPa (EPDM 50). Specific elongation decreased significantly with increasing wood sawdust content, from 976 ± 21% for EPDM 0 to 109 ± 32% for EPDM 50.

The incorporation of wood sawdust as a partial or full replacement for silica in EPDM composites significantly influences their mechanical properties. While the Young’s modulus increased with higher wood sawdust content, indicating enhanced stiffness, tensile strength and elongation at break decreased progressively. This suggests that although wood sawdust improves rigidity, it may compromise the material’s ability to withstand tensile forces and elongate before failure. These results highlight a trade-off between stiffness and flexibility when adjusting filler composition, emphasizing the need to optimize the balance between silica and natural fillers like wood sawdust for targeted applications. The wood sawdust improves stiffness but compromises the strength and flexibility due to weaker filler-matrix interactions and the rigid nature of the wood particles. This creates a trade-off, and the optimal balance depends on the desired application-more stiffness or more flexibility [56,57,58,59].

The following sections detail the effects of exposure to acidic environments—specifically, acetic acid solutions at concentrations of 10%, 20%, and 30%-on a series of EPDM-based composite materials (EPDM 0 to EPDM 50). We systematically analyze the changes in their physicochemical characteristics, mechanical performance, structural and morphological features, as well as thermal stability. This comprehensive evaluation aims to understand how varying the ratios of silica and wood sawdust fillers influences the composites’ resistance and durability under acidic conditions.

### 3.1. Swelling and Mass Loss Due to the Acetic Acid Exposure

**Figure 1:**
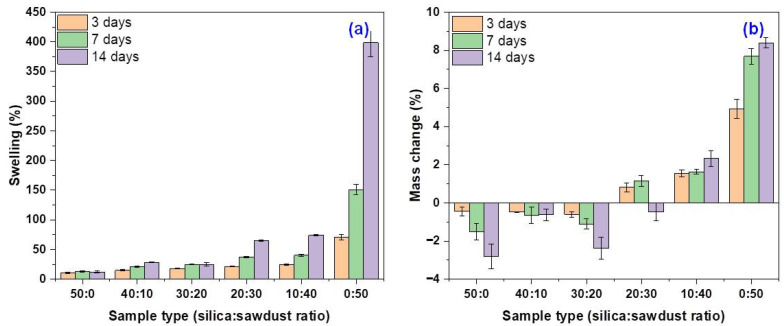
Swelling behavior (**a**) and mass change (**b**) of EPDM-based composites after immersion in 30% acetic acid solution for 3, 7, and 14 days.

Elastomers typically undergo swelling and mass changes when exposed to acidic environments. The extent of these effects depends on several factors, including the type of elastomer, the immersion time, and the acid concentration. In general, swelling increases with exposure time but tends to decrease as the acid concentration increases, since higher concentrations may limit solvent diffusion into the polymer network. At lower acid concentrations, elastomers often show greater swelling and mass variation, especially after prolonged exposure. In this study, the swelling behavior and mass changes of EPDM 0-EPDM 50 composites, following immersion in acetic acid solutions of 10%, 20%, and 30% for different time intervals (3, 7, and 14 days), are presented in Table 3 and Figure 1.

As shown in Table 3, the composites exhibited increased swelling with longer immersion times in the acid solution. Notably, even in the weakest concentration of 10% acetic acid, effective mass losses (negative values) were observed, suggesting the possible partial dissolution of both the elastomer matrix and the wood sawdust. Conversely, positive mass changes may indicate the potential binding or retention of acetic acid groups within the elastomer or filler, possibly through chemical interaction or absorption.

The EPDM 30–EPDM 50 composites stood out by exhibiting the highest swelling levels in all three acid solutions, along with mass increases, except for the EPDM 30 composite immersed in 30% acetic acid, which showed a mass loss after 14 days. This behavior, reduced swelling capacity paired with mass increase, can be attributed to the acetylation of the cellulose fibers in the wood sawdust. This chemical modification reduces the hygroscopic nature of the fibers and contributes to improved dimensional stability of the composite network [60,61]. Moisture absorption in cellulose-based fibers is minimized when good fiber–matrix adhesion is achieved. Although fully eliminating moisture uptake typically requires costly surface treatments [60], improvements can also be obtained through fiber modifications, such as acetylation or advanced three-step upgrading processes (hydrothermolysis, drying, and curing) [60,62]. The results in Table 3 and Figure 1 suggest that proper proportioning of fiber and elastomer prior to vulcanization may induce similar effects—such as improved compatibility and moisture resistance—without requiring separate fiber treatment steps, effectively achieving enhancement directly in the final composite product.

The immersion of rubber composites in acetic acid can lead to mass loss due to the acid’s corrosive nature and the leaching of plasticizers, fillers, and other low-molecular-weight additives from the rubber matrix [63]. Additionally, chemical degradation processes may occur. Acetic acid can interact with the polymer chains, promoting the formation of oxidized functional groups such as carbonyl and carboxyl groups. These modifications weaken both the intermolecular forces and covalent bonds within the polymer network, contributing to the breakdown of the rubber into smaller, more soluble fragments. As degradation progresses, mass loss increases, highlighting the role of acetic acid in facilitating the chemical deterioration of the elastomer matrix [64,65].

Moreover, the presence of wood sawdust as a natural, lignocellulosic filler may also contribute to mass loss. Wood is a hygroscopic, anisotropic material composed of cellulose (60–75%), lignin (20–30%), hemicelluloses (up to 30%), along with extractives and ash [66]. Upon exposure to acetic acid, the hemicelluloses-being more susceptible to acid hydrolysis than cellulose and lignin-undergo degradation [67,68]. During hydrolysis, water molecules cleave the hemicellulose chains into smaller, water-soluble fragments, which are subsequently leached from the fiber structure. This loss of material from the wood component leads to an overall decrease in the composite mass [69].

The observed swelling and mass variation in the EPDM-based composites exposed to acetic acid can be attributed to a combination of physical and chemical processes affecting both the elastomer matrix and the wood sawdust filler. While swelling generally increased with immersion time, the mass changes-both gains and losses-reflected complex interactions. Mass losses were primarily the result of chemical degradation, such as the leaching of low-molecular-weight components and acid-induced scission of polymer chains. In parallel, the hydrolysis of hemicelluloses in the wood sawdust contributed to further material loss. Conversely, certain formulations exhibited mass gain, likely due to acetylation-like reactions that improved the dimensional stability of the composite network and reduced the hydrophilicity of the wood fibers. These findings suggest that a proper balance between filler types and proportions can enhance the resistance of elastomeric composites in acidic environments, reducing degradability and potentially extending their service life.

### 3.2. Cross-Link Density Modification Due to the Acetic Acid Exposure

**Figure 2:**
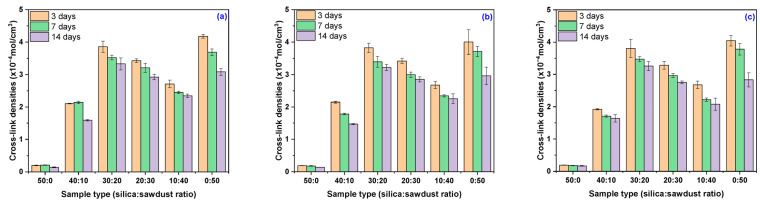
Variation of composite cross-link density as a function of immersion time and acetic acid concentration: (**a**) 10% acetic acid solution, (**b**) 20% acetic acid solution, and (**c**) 30% acetic acid solution.

The swelling behavior of polymers is strongly influenced by their cross-link density [70,71]. A lower cross-link density allows polymer chains to expand more easily, increasing the material’s ability to accommodate solvent molecules [70,72,73]. Conversely, a higher cross-link density restricts the mobility of the chains, reduces free volume within the network, and limits solvent uptake [70]. Typically, cross-linking density is adjusted by modifying the type and quantity of vulcanizing agents-such as accelerators or sulfur content-used during the curing process [70,74,75]. However, in the present study, the vulcanization conditions and agents were kept constant across all samples. Therefore, the observed variations in cross-link density are attributed solely to the nature and proportions of the fillers (silica and wood sawdust) incorporated into the EPDM matrix. The results of cross-link density behavior following immersion in acidic media are detailed in Table 4 and Figure 2, which reflect the influence of both filler composition and acid concentration on the chemical stability and network integrity of the composites.

As shown in Table 4 and Figure 2, all composites exposed for three days in an acidic environment, regardless of the solution concentration, exhibited an increase in cross-linking degree. After 7 days, the only composite that showed a slight decrease in cross-link density (below 5%) was EPDM 20. After 14 days, only one composite exhibited an increase in cross-link density compared to the initial values (Table 2); however, compared to the first exposure period, this parameter actually decreased. The most stable composites (those whose cross-link density changed by only 0.5% to 4% as immersion time and acetic acid concentration increased from 10% to 30%) were EPDM 30 (0.51%) and EPDAM 20 (3.69%). The other composites suffered changes greater than 5% (EPDM 0 at 19.43%, EPDM 10 at 11.13%, EPDM 40 at 10.3%, and EPDM 50 at 5.39%). This observation correlates with the results from the swelling and mass loss experiments, where EPDM 20 showed decreased swelling and increased mass, likely due to reduced hygroscopicity of sawdust caused by acetylation of hydroxyl groups and ester bond formation [60,62]. During aging, the main molecular chain of peroxide-cured EPDM undergoes scission, resulting in the formation of double bonds and C=O structures. Additionally, peroxide-cured EPDM experiences a cross-linking reaction in the later stage of aging, forming an ether-cross-linked network that helps restore the degraded cross-linking structure [76]. These findings are also supported by changes in mechanical properties, where an increase in elongation at break is associated with molecular chain scission, while a decrease in elongation indicates polymer chain cross-linking [77,78]. In the presence of acetic acid, initial chain scission occurs due to chemical degradation, followed by increased cross-linking involving previously dangling chains in the rubber network, ultimately leading to an overall increase in cross-link density [76].

### 3.3. Mechanical Characteristics Modification Due to the Acetic Acid Exposure

Thin fibers as flax, jute or hemp are considered the bests for improvements in tensile, bending strength as well as modulus; however, fillers derived from wood processing can be effectively used to modulate dimensional stability (swelling) and stiffness [60].

**Figure 3:**
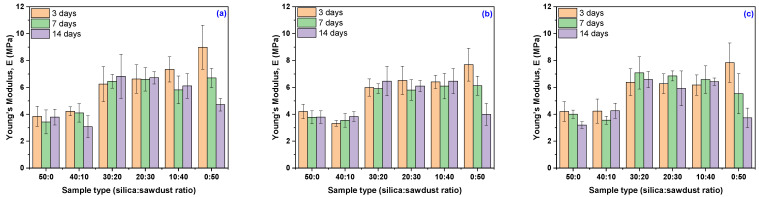
Variation of composites’ Young’s modulus, *E* (MPa), as a function of acetic acid concentration and immersion time: (**a**) 10% acetic acid in solution, (**b**) 20% acetic acid in solution, (**c**) 30% acetic acid in solution.

From Table 5 and Figure 3, it can be observed that the composites with a small difference between wood sawdust and silica content (EPDM 20 and EPDM 30) exhibit the greatest stability. These composites underwent minor changes with increasing exposure time to the acidic solution, with EPDM 20 showing a change in strength from −7% to +1.55%, and EPDM 30 from −4.3% to −3.02%. Correspondingly, their mechanical strength increased from 1.55 to 5.42 for EPDM 20, and from −3.02 to −0.95 for EPDM 30.

It can also be observed that, although most composites experienced significant changes in stiffness, the Young’s modulus remained relatively stable (between 5 and 6 MPa) in composites with either the lowest (EPDM 0 and EPDM 10) or highest (EPDM 50) wood sawdust content. The typical Young’s modulus of EPDM rubber ranges from 2 to 10 MPa, depending on filler content (e.g., carbon black) and formulation. Values around 6–7 MPa are considered very good for many applications where the elastomer is reinforced with conventional fillers [18].

As expected, replacing silica with wood sawdust from 10 phr to 50 phr led to an increase in Young’s modulus, with the composites becoming less stiff due to the inherent properties of the fillers and the rubber-filler interactions. Exposure to acidic solutions highlighted the specific silica-to-sawdust ratios for which Young’s modulus remains stable-an observation that correlates with both the degree of crosslinking and swelling behavior of the samples. These findings support the previous assumption that acetylation of hydroxyl groups in cellulose occurred, which helped the added sawdust maintain the Young’s modulus at approximately 6 MPa [18,79].

**Figure 4:**
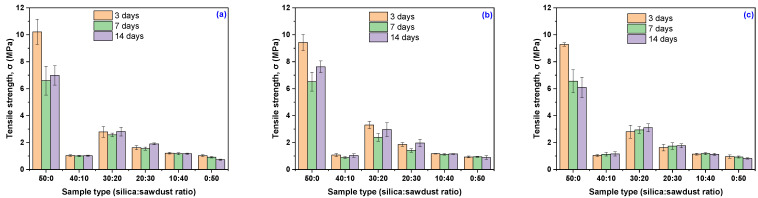
Variation of composites’ Tensile strength, σ (MPa), as a function of acetic acid concentration and immersion time: (**a**) 10% acetic acid in solution, (**b**) 20% acetic acid in solution, (**c**) 30% acetic acid in solution.

The results regarding the behavior of the composites in acidic environments in terms of tensile strength are presented in Table 6 and Figure 4. As observed, EPDM 20 and EPDM 30 exhibit the greatest stability over time across all three acidic solutions. Although their tensile strength values decreased compared to the initial measurements, they remained consistently low and stable during exposure. Conversely, the composites with higher wood sawdust content (EPDM 40 and EPDM 50) maintained tensile strength values relatively close to the initial ones after 3 to 14 days of immersion in any of the acidic solutions. These findings support the initial hypothesis that incorporating wood sawdust as a filler in EPDM-based composites enhances resistance to chemical degradation in acidic environments, primarily due to the reduced hydrophilicity of the sawdust following chemical modifications.

**Figure 5:**
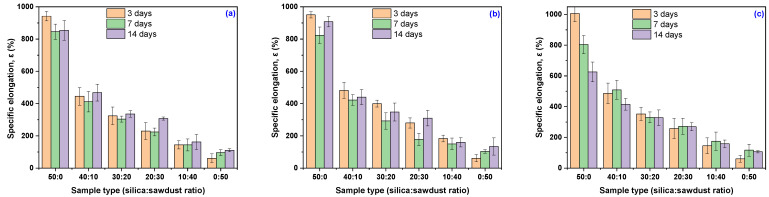
Composites elongation at break, ε (%), as a function of acetic acid concentration and immersion time: (**a**) 10% acetic acid in solution, (**b**) 20% acetic acid in solution, (**c**) 30% acetic acid in solution.

As shown in Table 7 and Figure 5, the specific elongation of EPDM 20 and EPDM 30 composites increased after just three days of exposure to acetic acid solutions at 20% and 30%, with these elevated values maintained even after 14 days. In the 10% acetic acid solution, the specific elongation also increased compared to the initial value, but only after 14 days of immersion. In contrast, samples with higher wood sawdust content (EPDM 40 and EPDM 50), as well as the composite without any wood sawdust (EPDM 0), were negatively affected by acetic acid exposure, showing degradation even at the lowest acid concentration and shortest exposure time.

The mechanical characterization of the EPDM composites revealed that formulations with balanced ratios of silica and wood sawdust fillers (EPDM 20 and EPDM 30) exhibited the best overall stability when exposed to acidic environments. These composites maintained relatively stable Young’s modulus and tensile strength values over prolonged exposure to acetic acid, indicating resistance to stiffness and strength degradation. Additionally, their specific elongation improved or remained stable, suggesting enhanced flexibility and durability under acidic conditions. Conversely, composites with either very low or very high wood sawdust content (EPDM 0, EPDM 10, EPDM 40, and EPDM 50) were more susceptible to mechanical deterioration, showing significant reductions in tensile strength and elongation. These findings highlight the critical role of filler composition in optimizing the mechanical performance and acid resistance of EPDM-based composites.

The addition of wood sawdust combined with the reduction of silica in the mixture leads to a decrease in elongation at break, which can be attributed to increased stress concentration and restricted mobility of the polymer chains [80]. Excessive sawdust content, along with poor dispersion within the mixture, further contributes to this decline. Dispersion involves more than simply mixing and distributing filler particles in the rubber matrix; it requires an energy-intensive process to break down agglomerates, increase surface area, and achieve a uniform distribution at the molecular level. Effective dispersion also ensures strong filler–matrix interactions, with each particle adequately coated by the rubber matrix [79,80,81,82].

The mechanical properties of EPDM composites-such as tensile strength, elongation at break, and Young’s modulus-are critical indicators of their durability and structural integrity in chemically aggressive environments. In this study, maintaining mechanical performance after exposure to acetic acid demonstrates the composites’ potential for long-term use in applications requiring resistance to acidic corrosion, such as gaskets, seals, and industrial linings. The optimized filler ratio, particularly in EPDM 20 and EPDM 30, ensures a balance between stiffness and elasticity, contributing to extended service life and reduced material failure in corrosive settings.

### 3.4. Morphological Modifications due to the Acetic Acid Exposure

**Figure 6:**
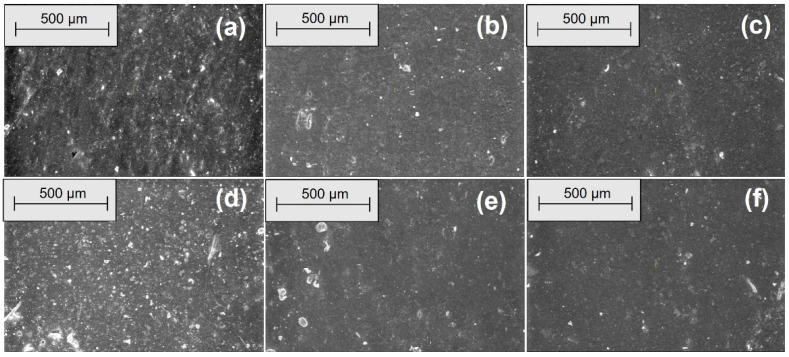
SEM microphotographs of surfaces from transverse cuts at 100× magnification: (**a**) EPDM 0, (**b**) EPDM 10, (**c**) EPDM 20, (**d**) EPDM 30, (**e**) EPDM 40, (**f**) EPDM 50.

**Figure 7:**
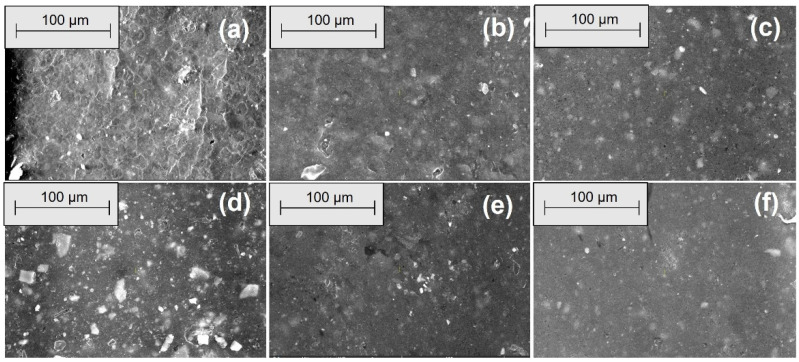
SEM microphotographs of surfaces from transverse cuts at 500× magnification: (**a**) EPDM 0, (**b**) EPDM 10, (**c**) EPDM 20, (**d**) EPDM 30, (**e**) EPDM 40, (**f**) EPDM 50.

**Figure 8:**
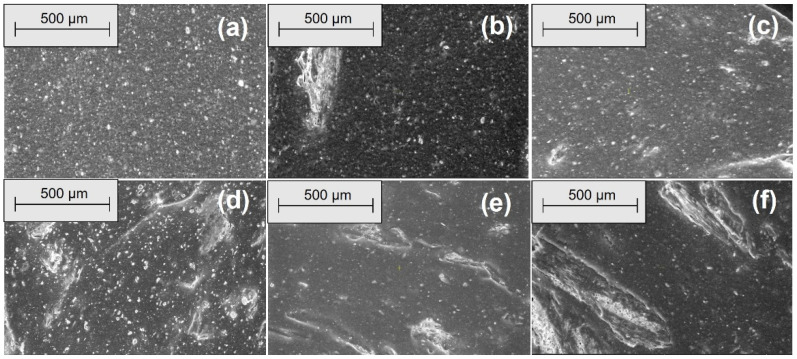
SEM microphotographs of surfaces from longitudinal cuts at 100× magnification: (**a**) EPDM 0, (**b**) EPDM 10, (**c**) EPDM 20, (**d**) EPDM 30, (**e**) EPDM 40, (**f**) EPDM 50.

**Figure 9:**
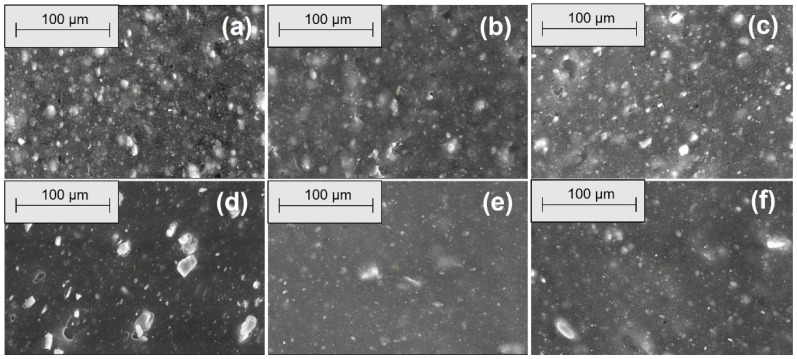
SEM microphotographs of surfaces from longitudinal cuts at 500× magnification: (**a**) EPDM 0, (**b**) EPDM 10, (**c**) EPDM 20, (**d**) EPDM 30, (**e**) EPDM 40, (**f**) EPDM 50.

Figure 6, Figure 7, Figure 8 and Figure 9 show the SEM microphotographs of the composite samples (EPDM 0-EPDM 50) before exposure to acidic environment. The images were taken at magnifications of 100× and 500× on surfaces obtained from transverse and longitudinal cuts, respectively.

As shown in Figure 6, the SEM micrographs of the composites’ transverse surfaces at 100× magnification show that EPDM 0 (silica only) has a smooth and uniform surface. As wood sawdust content increases (EPDM 10 to EPDM 50), filler particles become more apparent. Composites EPDM 20 and EPDM 30 demonstrate better dispersion with fewer agglomerates, while EPDM 40 and EPDM 50 display more filler clusters and voids, indicating less uniform filler distribution. Figure 7 shows SEM micrographs of the composites’ transverse surfaces at 500× magnification. EPDM 0 (a) presents a smooth surface with minimal visible particles. As wood sawdust content increases (EPDM 10 to EPDM 50), filler particles become more noticeable. EPDM 20 (c) and EPDM 30 (d) demonstrate relatively uniform filler dispersion with fewer agglomerates, whereas EPDM 40 (e) and EPDM 50 (f) exhibit more particle clusters and voids, indicating poorer filler distribution and potential weak points in the matrix.

Figure 8 shows SEM micrographs of the composites’ longitudinal surfaces at 100× magnification. EPDM 0 (a) exhibits a smooth and homogeneous surface with minimal texture. With increasing wood sawdust content (EPDM 10 to EPDM 50), filler particles and fiber-like structures become more prominent. EPDM 20 (c) and EPDM 30 (d) show well-dispersed fillers with fewer agglomerates, while EPDM 40 (e) and EPDM 50 (f) display larger filler clusters and voids, indicating less uniform distribution and potential weak points in the composite matrix. Figure 9 presents SEM micrographs of the composites’ longitudinal surfaces at 500× magnification. EPDM 0 (a) displays a relatively smooth surface with small filler particles evenly dispersed. As the wood sawdust content increases from EPDM 10 to EPDM 50 (b–f), the presence of filler particles becomes more evident. EPDM 20 (c) and EPDM 30 (d) maintain good filler dispersion with fewer agglomerates, whereas EPDM 40 (e) and EPDM 50 (f) show more pronounced filler clusters and voids, indicating less uniform filler distribution and potential areas of weakness within the composite matrix.

**Figure 10:**
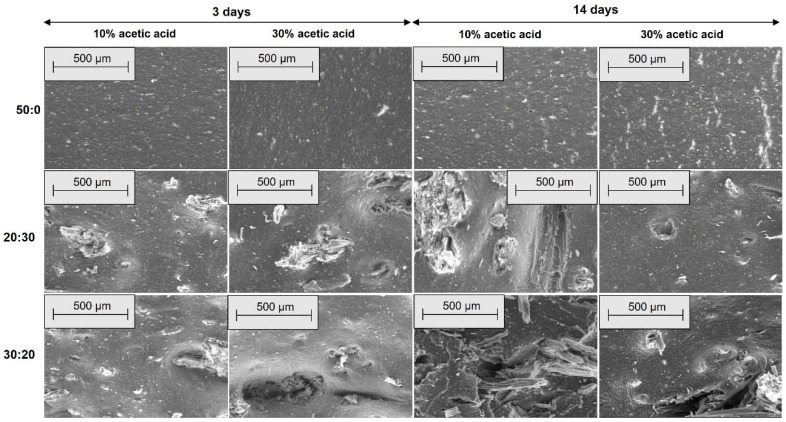
SEM microphotographs of surfaces from transverse cuts at 100× magnification for EPDM 0 (50:0), EPDM 20 (20:30) and EPDM 30 (30:20) after acetic acid exposure.

**Figure 11:**
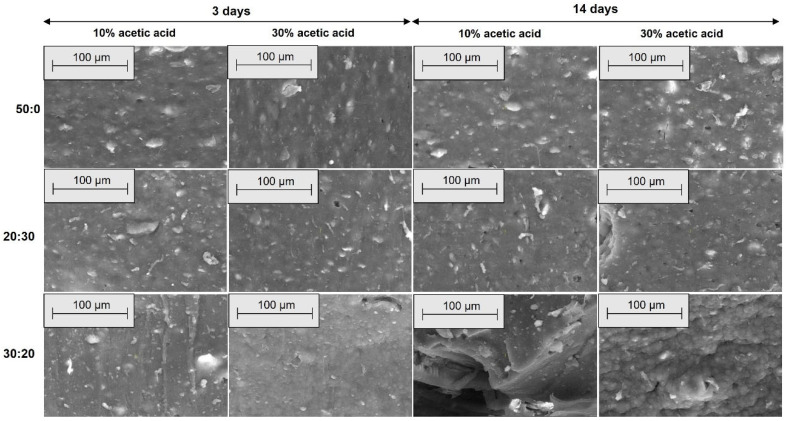
SEM microphotographs of surfaces from transverse cuts at 500× magnification for EPDM 0 (50:0), EPDM 20 (20:30) and EPDM 30 (30:20) after acetic acid exposure.

**Figure 12:**
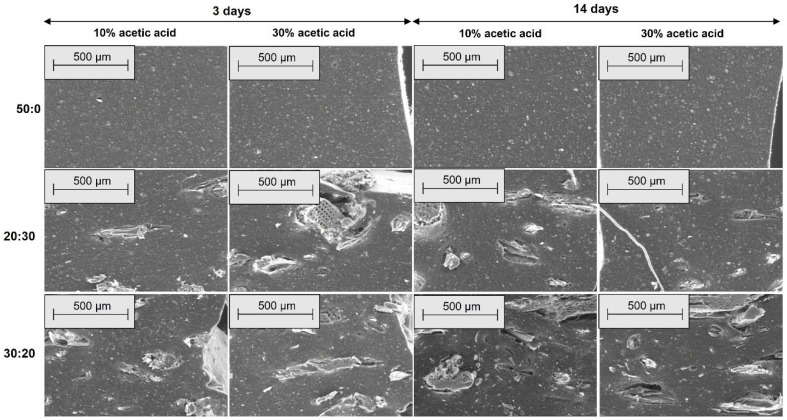
SEM microphotographs of surfaces from longitudinal cuts at 100× magnification for EPDM 0 (50:0), EPDM 20 (20:30) and EPDM 30 (30:20) after acetic acid exposure.

**Figure 13:**
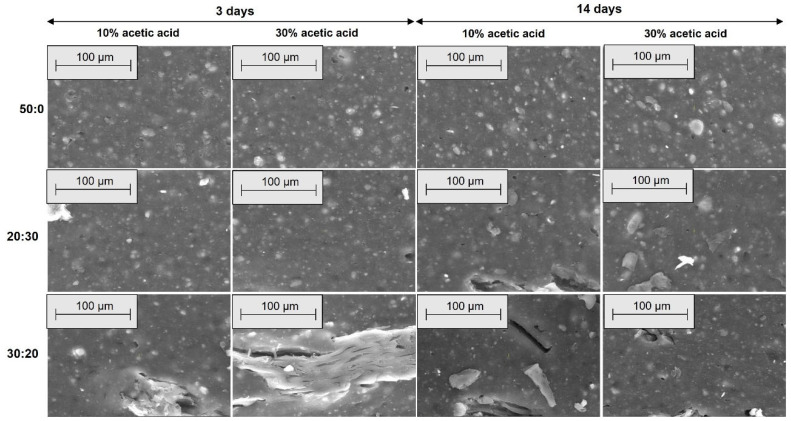
SEM microphotographs of surfaces from longitudinal cuts at 500× magnification for EPDM 0 (50:0), EPDM 20 (20:30) and EPDM 30 (30:20) after acetic acid exposure.

Figure 10, Figure 11, Figure 12 and Figure 13 present SEM images of these composites, compared to the EPDM 0 composite, which does not contain wood sawdust.

Figure 10, Figure 11, Figure 12 and Figure 13 show SEM micrographs (100× and 500× magnification) of EPDM-based composite surfaces (transverse and longitudinal cuts) after immersion in 10% and 30% acetic acid solutions for 3 and 14 days. The EPDM 0 (50:0) samples, containing only silica as filler, retain relatively smooth and uniform surfaces with minimal degradation signs across both acid concentrations and exposure durations. This indicates limited interaction between the matrix and the acidic medium. In contrast, EPDM 20 (20:30) and EPDM 30 (30:20) composites, which incorporate wood sawdust, exhibit noticeable surface roughening, filler pull-out, and the development of voids and cracks. These features are more pronounced with increasing acid concentration and immersion time, particularly at 14 days in 30% acetic acid. Despite these changes, the degradation is not uniformly severe. EPDM 30 samples display more compact and interconnected filler–matrix regions, suggesting better structural integrity compared to EPDM 20. This could be attributed to improved filler dispersion and potential chemical modifications of the sawdust (e.g., acetylation), which reduce hydrophilicity and enhance acid resistance. Overall, these observations support previous mechanical and swelling data, confirming that appropriate silica-sawdust ratios (e.g., 30:20) can contribute to enhanced acid durability of EPDM composites through improved interfacial interactions.

The SEM micrographs reveal that filler distribution, interfacial adhesion, and surface integrity of the EPDM composites vary significantly with sawdust content and acid exposure. Prior to immersion, EPDM 20 and EPDM 30 composites exhibit better filler dispersion and fewer agglomerates compared to those with higher sawdust content (EPDM 40 and EPDM 50), which show visible clusters and voids. After acetic acid exposure, EPDM 0 maintains a smooth surface, indicating high chemical stability. In contrast, EPDM 20 and EPDM 30 show moderate degradation, such as fiber pull-out and matrix erosion, especially after prolonged exposure to concentrated acid. However, they retain better structural integrity compared to composites with higher sawdust content, suggesting that an optimal wood-silica ratio enhances filler-matrix interaction and improves resistance to chemical attack [60,61].

### 3.5. Structural Modifications due to the Acetic Acid Exposure

Wood sawdust mainly consists of cellulose (38–50%), lignin (15–25%) and hemicelluloses (23–32%) [83,84,85]. Chemically, it contains approximately 60.8% carbon, 33.8% oxygen, 5.2% hydrogen, and 0.9% nitrogen. Functional groups such as hydroxyl, carbonyl, ether, carboxyl, and ester (originating from cellulose, lignin, and hemicelluloses) can be identified in FTIR-ATR spectra [86,87,88,89,90,91].

**Figure 14:**
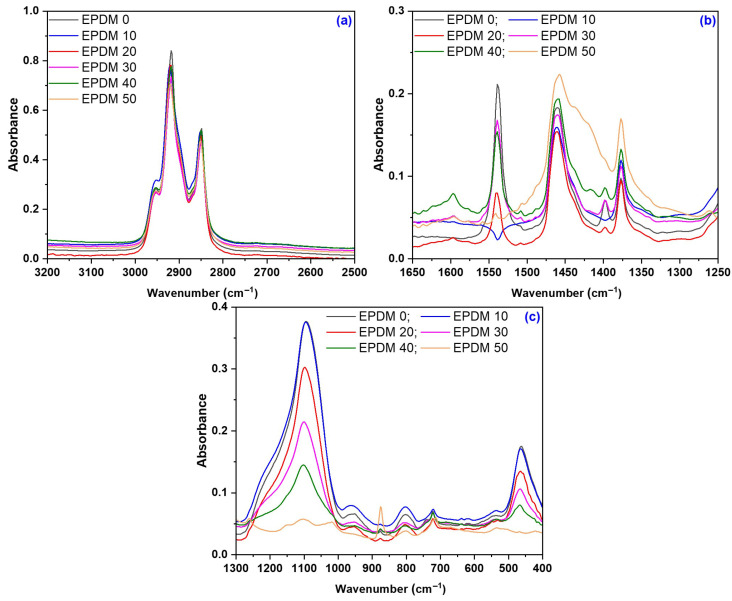
FTIR-ATR spectra of EPDM 0 to EPDM 50 composites in the regions: (**a**) 2500–3300 cm^−1^, (**b**) 1250–1700 cm^−1^, and (**c**) 400–1300 cm^−1^.

**Figure 15:**
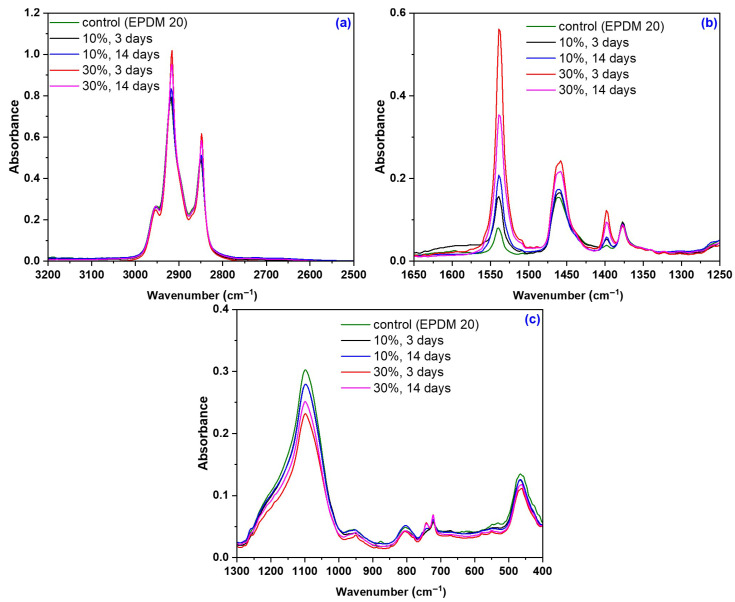
FTIR-ATR spectra of EPDM 20 composites after immersion in acetic acid solutions: (**a**) 2500-3300 cm^−1^, (**b**) 1250-1700 cm^−1^, and (**c**) 400-1300 cm^−1^.

**Figure 16:**
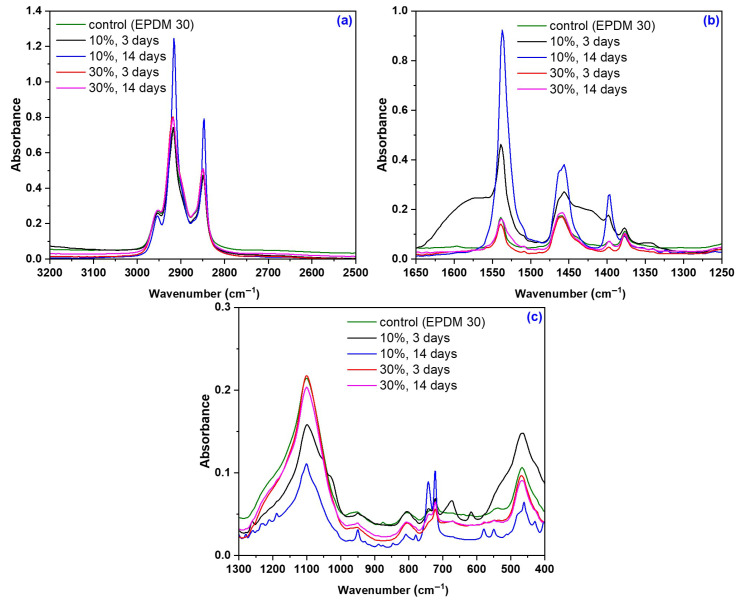
FTIR-ATR spectra of EPDM 30 composites after immersion in acetic acid solutions: (**a**) 2500–3300 cm^−1^, (**b**) 1250–1700 cm^−1^, and (**c**) 400–1300 cm^−1^.

Figure 14 shows the FTIR-ATR spectra of EPDM 0-EPDM 50 composites before immersion in acetic acid, with spectral details highlighted in the ranges 2500–3300 cm^−1^, 1250–1700 cm^−1^ and 400–1300 cm^−1^. Figure 15 and Figure 16 presents the spectra of EPDM 20 and EPDM 30 after the immersion in 10–30% acetic acid solutions. The band assignments for these spectra are summarized in Table 8.

The bonding between the EPDM rubber and wood sawdust is primarily due to the formation of cellulose radicals in the pyranose ring during vulcanization. Wood sawdust contains polymers as cellulose, hemicelluloses and lignin, which have many active functional groups susceptible to chemical reactions [92]. Cellulosic materials consist of both crystalline and amorphous domains, with their physical and chemical properties and reactivity strongly influenced by the molecular arrangement relative to each other and to the fiber axis. Most chemical reactions occur in the amorphous regions and on the surface of crystallites, while intra-crystalline regions remain largely unaffected [93]. The presence of amorphous cellulose is confirmed by absorption bands between 2940–2840 cm^−1^, particularly at 2920 and 2850 cm^−1^, attributed to asymmetric and symmetric C-H stretching vibrations in methyl and methylene groups [93]. These bands are characteristic of the saturated hydrocarbon backbone from EPDM and the wood polymers cellulose, lignin, and hemicellulose [94]. Additionally, absorption bands at 2970–2920 cm^−1^ indicate the presence of methoxy groups (-OCH_3_), characteristic of lignin [92]. Bands in the 1665–1660 cm^−1^ region, especially in samples with a higher sawdust content, correspond either to the absorbed water in cellulose [90], or lignin aromatic skeletal vibrations [93]. Bands at 1550–1520 cm^−1^ arise from conjugated C-O stretching and aromatic skeletal vibrations of lignin and wood sawdust [92,95,96]. The 1480–1360 cm^−1^ region corresponds to hydroxil (-OH) and methylene (-CH_2_-) groups in aromatic nuclei of wood sawdust, as well as C-H deformation in lignin, cellulose, hemicelluloses and carbohydrates [96]. The 1480–1430 cm^−1^ band is known as “crystallinity band” [97], while methyl groups produce symmetrical and asymmetrical bending bands at 1410–1390 cm^−1^ and 1480–1430 cm^−1^, respectively [98]. Low-intensity bands between 1320–1220 cm^−1^, are assigned to C-H vibration in cellulose, C1-O vibration in syringyl ring and C-O stretching in lignin and xylan [96]. Bands at 1140–1020 cm^−1^ are associated with -O-C vibration in cellulose and hemicelluloses, aromatic skeletal, C-O stretching vibrations in cellulose and hemicelluloses and C-O deformation from primary alcohols of lignin, confirming the presence of wood sawdust in composite [91,92,93,94]. Furthermore, absorption bands between 650–900 cm^−1^, related to C-H deformation in cellulose, also indicate aromatic nuclei from sawdust [92,96].

In all composites, the EPDM is identified by absorption bands around 1456 cm^−1^ (-CH_2_ scissoring vibrations), 1377 cm^−1^ (C-H bending vibration of -CH_3_ groups), 722 cm^−1^ (-CH_2_ rocking vibrations), and the intense bands at 2920 and 2850 cm^−1^ (asymmetric and symmetric C-H stretching vibrations) [86,99,100,101]. Additionally, all spectra show absorptions related to the vulcanizing agent benzoyl peroxide, including a weak band at 950–800 cm^−1^ due to O-O stretching and a band between 1300-1050 cm^−1^ from C-O stretching, highlighting the dominance of C-O bonds over the O-O bonds [69].

In Figure 14, it can be observed that the spectra of the EPDM 10 and EPDM 50 do not prominently display the bands typically associated with the aromatic skeletal vibrations from lignin, nor the C=O stretches in non-conjugated ketones, carbonyls, and in ester groups usually found around 1539 cm^−1^ [88]. Additionally, the characteristic bands for –CH_2_ stretching and bending vibrations and C-O stretching, linked to the crystalline structure of the cellulose and typically found at 1398 cm^−1^ [89,90,91] and 1098 cm^−1^, respectively, are not clearly visible. However, in the spectra of EPDM 50, there is a noticeable intensification of the band at 890 cm^−1^, which is associated with O-O stretching vibration [91].

In the spectra of EPDM 20 and EPDM 30 shown in Figure 15 and Figure 16, several changes can be observed which, although not indicating significant structural modifications, align with the mechanical test results. The chemical modification of the wood sawdust components after immersion in acetic acid is primarily due to substitution reaction in which hydrophilic hydroxyl groups (-OH) are replaced by a hydrophobic acetyl group (CH_3_CO-). This acetylation process involves the nucleophilic attack by the lone electron pair of the alcoholic or phenolic (-OH) group in the sawdust on the carbonyl group of acetic acid, resulting in the formation of ester bonds and the release of acetic acid. Consequently, the number of hydroxyl groups decreases while the number of acetyl groups increases [102]. The vibration bands corresponding to cellulose and methoxy group (-OCH_3_), characteristic of lignin, in the 2920-2840 cm^−1^ region remain largely unchanged after acid treatment, likely because the lignin matrix protects cellulose from direct acid contact [69].

The presence of acetyl groups formed during acetylation of sawdust is confirmed by bands in the 1450–1350 cm^−1^ and 1250–1220 cm^−1^ regions, particularly near to 1435, 1375 and 1240 cm^−1^. These are associated with C-O carbonyl stretching and CH_3_ symmetric and asymmetric deformation vibrations [103,104,105,106]. These bands indicate esterification reaction, specifically the substitution of -OH groups with acetyl groups. Additionally, they correspond to asymmetric C-O stretching in the grafted ester group (C-O-C=O), C-C and C-O bond stretching, and the in-plane =C–H deformation vibrations of aromatic ring in lignin [102,107,108]. An increased intensity of these bands suggests a higher degree of substitution, typically accompanied by reduced band intensity near 1082 and 1014 cm^−1^, indicative of fewer hydroxyl groups in the structure [103].

### 3.6. TGA/DSC Modifications due to the Acetic Acid Exposure

The resulting thermograms were processed using Proteus analysis software (version 6.1.0/2019) integrated with the STA 409 PC thermal analysis system [109].

**Figure 17:**
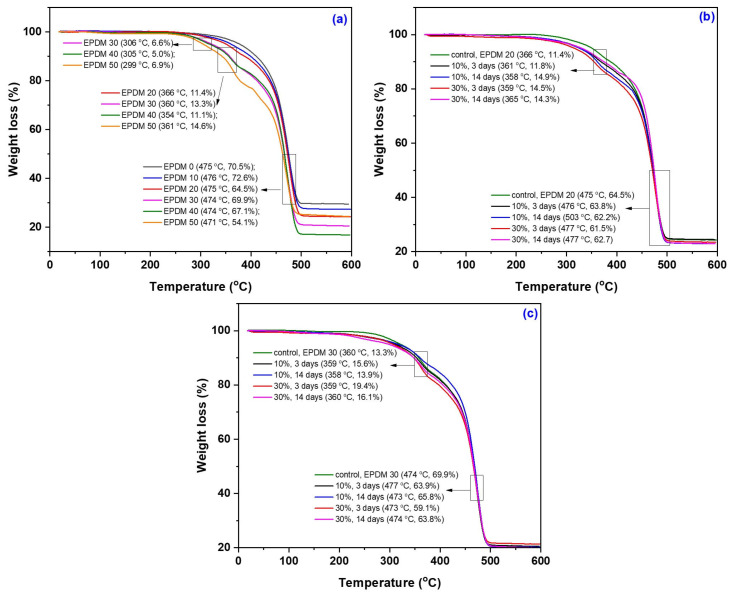
Thermogravimetry analysis (TGA) curves of EPDM 0 to EPDM 50 (**a**), EPDM 20 and EPDM 30 composites after immersion in acetic acid solutions (**b**,**c**).

**Figure 18:**
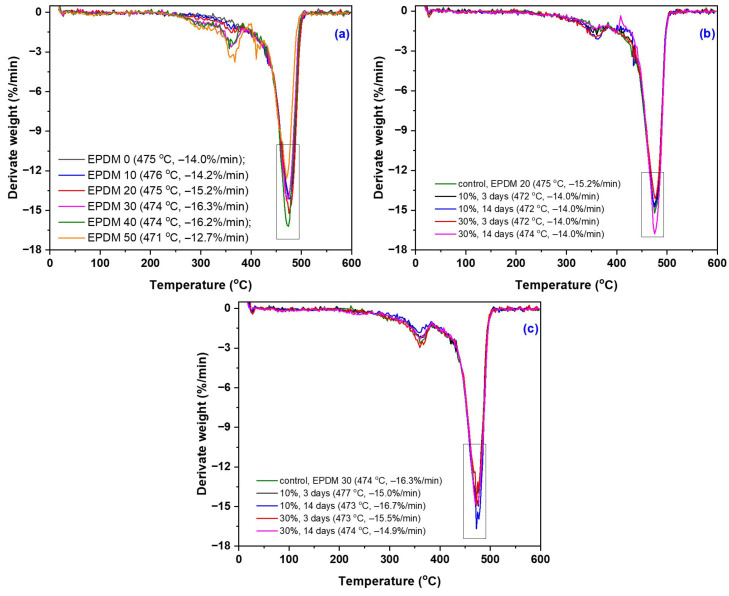
Differential weight loss (DTG) curves of EPDM 0 to EPDM 50 (**a**), EPDM 20 and EPDM 30 composites after immersion in acetic acid solutions (**b**,**c**).

**Figure 19:**
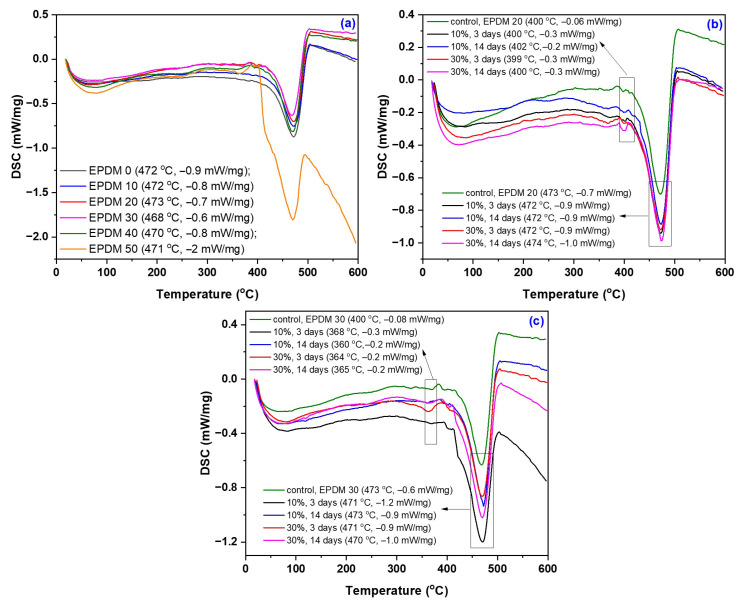
Differential Scanning Calorimetry (DSC) curves of EPDM 0 to EPDM 50 (**a**), EPDM 20 and EPDM 30 composites after immersion in acetic acid solutions (**b**,**c**).

TGA thermograms, their derivative curves (DTG), and DSC curves of the EPDM 0–50 composites are presented in Figure 17, Figure 18 and Figure 19, with corresponding data summarized in Table 9. The results include both the composites prior to immersion in acetic acid solutions (10–30%) and the EPDM 20 and EPDM 30 composites after acid exposure.

The TGA results of the EPDM composites (EPDM 0–EPDM 50) revealed two main stages of thermal degradation [110]. The first stage, occurring between approximately 200 °C and 400 °C, corresponds primarily to the degradation of wood sawdust components (hemicellulose and cellulose). The second degradation stage, typically observed between 400 °C and 550 °C, is associated with the decomposition of the EPDM rubber matrix and residual lignin. Composites with higher wood sawdust content (EPDM 40 and EPDM 50) exhibited an earlier onset of thermal degradation, due to the lower thermal stability of lignocellulosic materials. On the other hand, EPDM 0 and EPDM 10, which contain only or mostly silica, showed a delayed onset of thermal degradation, indicating higher thermal stability [111].

After immersion in acetic acid (10 and 30%), EPDM 20 and EPDM 30 composites demonstrated minor changes in thermal stability. Slight reductions in onset degradation temperatures were noted, but no significant structural breakdown occurred, supporting the FTIR and mechanical test results. The residual mass increased slightly for acid-treated samples, suggesting potential cross-linking or chemical stabilization (e.g., acetylation) of sawdust components.

Overall, the TGA results confirm that moderate wood sawdust content (EPDM 20 and EPDM 30) maintains good thermal stability even after exposure to acidic environments, making them suitable candidates for applications requiring chemical resistance and dimensional stability.

The thermal analysis revealed that EPDM 10 and EPDM 20 composites exhibited a single main decomposition stage, corresponding to the thermal degradation of the EPDM matrix. The EPDM 30 composite showed two distinct decomposition stages, while EPDM 40 and EPDM 50 exhibited three stages, indicating the progressive degradation of both the polymer matrix and the lignocellulosic fillers. A single degradation stage is typically indicative of good miscibility between the polymer and filler components [112]. Notably, after immersion in acetic acid solutions, both EPDM 20 and EPDM 30 composites presented a single thermal decomposition stage, suggesting improved compatibility and potential chemical stabilization of the sawdust within the rubber matrix following acid treatment.

## 4. Conclusions

Composite materials based on EPDM and hybrid fillers-comprising silica and wood sawdust in controlled ratios (50:0, 40:10, 30:20, 20:30, 10:40, 0:50 phr)-were evaluated for their resistance to chemical degradation in a mildly acidic yet strongly corrosive environment (acetic acid solutions ranging from 10% to 30%).

The study demonstrates that incorporating wood sawdust as a partial replacement for silica in EPDM rubber composites significantly influences their mechanical, morphological, chemical, and thermal behavior-especially under acidic exposure. Among the tested formulations, EPDM 20 (20% sawdust/30% silica) and EPDM 30 (30% sawdust/20% silica) consistently exhibited the most balanced and stable performance across all test categories.

Mechanically, these two composites maintained stable tensile strength, minimal changes in cross-link density, and preserved Young’s modulus after prolonged immersion in acetic acid solutions. The elongation at break improved or remained stable in these samples, indicating a reduction in chain scission and effective reinforcement due to good filler–matrix interactions.

SEM analyses confirmed better dispersion and lower filler agglomeration in EPDM 20 and EPDM 30 compared to other formulations, correlating with their mechanical stability. Poor dispersion and visible voids were evident in composites with either too little or too much sawdust (e.g., EPDM 0, 10, 50), leading to more significant property degradation.

FTIR-ATR results indicated chemical interactions between EPDM and the wood sawdust. The appearance of absorption bands characteristic of ester groups confirmed acetylation of hydroxyl groups during acid exposure. This chemical modification contributed to the reduced hydrophilicity of sawdust, explaining the improved dimensional stability and reduced swelling in the composites. The FTIR data also supported the presence of key functional groups from cellulose, lignin, and hemicelluloses, indicating that sawdust remained chemically active and compatible within the matrix.

Thermogravimetric analysis (TGA) revealed one main decomposition stage in EPDM 20 and 30, before and after acid immersion-suggesting good miscibility and thermal stability. In contrast, composites with higher sawdust content exhibited two or three decomposition stages, attributed to the sequential breakdown of lignocellulosic components. These differences emphasize the importance of optimizing filler ratios to ensure thermal coherence within the composite.

Overall, the results confirm that the chemical modification of sawdust under acidic conditions, combined with its partial replacement of silica, can lead to EPDM composites with enhanced acid resistance, stable mechanical performance, and satisfactory thermal properties-provided the filler dispersion and ratio are properly controlled. Composites like EPDM 20 and EPDM 30 represent optimal formulations for applications requiring resistance to chemical aging and mechanical stability in acidic environments.

## Figures and Tables

**Table 1:** Recipes for EPDM composite preparation.

Mixture Code/Recipe (phr)	EPDM 0	EPDM 10	EPDM 20	EPDM 30	EPDM 40	EPDM 50
EPDM Keltan 512 (EPDM-ENB, ~60% ethylene)	100	100	100	100	100	100
Filler (silica)	50	40	30	20	10	0
Filler (wood sawdust)	0	10	20	30	40	50
Plasticizer, paraffinic oils	40	40	30	30	20	25
ZnO	4.5	4.5	4.5	4.5	4.5	4.5
Stearine	1.5	1.5	1.5	1.5	1.5	1.5
PEG 4000	3	3	3	3	3	3
TiO2	4	4	4	4	4	4
Antioxidants, IPPD (4010)	1	1	1	1	1	1
Vulcanizing accelerator, dicyclopentadiene (DCP)	3	3	3	3	3	3
Vulcanizing agent, dicumyl peroxide (DCP)	1.5	1.5	1.5	1.5	1.5	1.5

**Table 2:** Characterization parameters of control samples, including rubber-filler interaction parameter (V_ro_/V_rf_), solvent uptake in toluene (mL/g), cross-link density (×10^−4^ mol/cm^3^), Young’s modulus (MPa), tensile strength (MPa), and specific elongation (%).

Sample Code	V_rf_	V_ro_/V_rf_	Solvent Uptake	Cross-Link Density	Young’s Modulus (E)	Tensile Strength (σ)	Specific Elongation (ε)
EPDM 0	-		1.505 ± 0.137	0.1729 ± 0.0062 ^e^	5.03 ± 0.71 ^cd^	6.25 ± 0.19 ^a^	976 ± 21 ^a^
EPDM 10	0.2872	0.4500	0.630 ± 0.043	1.6168 ± 0.0206 ^d^	4.12 ± 0.52 ^d^	3.54 ± 0.57 ^b^	584 ± 72 ^b^
EPDM 20	0.3402	0.3799	0.446 ± 0.005	3.6900 ± 0.1649 ^a^	6.72 ± 1.19 ^bc^	3.08 ± 0.45 ^b^	330 ± 25 ^c^
EPDM 30	0.3449	0.3748	0.391 ± 0.028	2.8949 ± 0.12978 ^b^	6.92 ± 0.87 ^b^	1.60 ± 0.15 ^c^	246 ± 36 ^d^
EPDM 40	0.2991	0.4322	0.537 ± 0.034	2.2149 ± 0.13008 ^c^	8.13 ± 0.71 ^ab^	1.50 ± 0.05 ^c^	298 ± 43 ^cd^
EPDM 50	0.2948	0.4384	0.486 ± 0.027	2.1974 ± 0.10904 ^c^	9.12 ± 1.47 ^a^	1.02 ± 0.05 ^c^	109 ± 32 ^e^
*F* value				582.9998	18.56968	189.03578	290.88586
*p* value				<0.0001	<0.0001	<0.0001	<0.0001

The values represent the averages of three samples. Values within each column bearing different lowercase superscripts indicate significant differences (*p* ≤ 0.05). Statistical analysis was performed using one-way ANOVA, with test results reported as F-statistic (F-value) and *p*-value. A *p*-value less than 0.05 indicates significant differences between sample means. For this analysis: number of groups (k) = 6, total samples (n) = 30, degrees of freedom for numerator (df_1_) = 5, degrees of freedom for denominator (df_2_) = 24, and the critical F-value = 2.6207.

**Table 3:** Swelling (%) and mass change (%) of EPDM-based composites after immersion in acetic acid solutions (10%, 20%, and 30%) for 3, 7, and 14 days.

Sample Code	3 Days		7 Days		14 Days	
	Swelling	Mass Change	Swelling	Mass Change	Swelling	Mass Change
Acetic acid concentration 10%						
EPDM 0	+13.57 ± 1.51	−0.48 ± 0.29	+12.46 ± 1.69	−1.39 ± 0.39	+12.38 ± 0.43	−1.76 ± 0.31
EPDM 10	+16.17 ± 2.11	−0.36 ± 0.17	+21.11 ± 1.46	−0.69 ± 0.32	+29.89 ± 0.88	−0.52 ± 0.19
EPDM 20	+21.73 ± 1.16	−0.43 ± 0.20	+24.73 ± 0.64	−0.75 ± 0.36	+28.82 ± 1.01	−1.23 ± 0.33
EPDM 30	+23.28 ± 1.39	+0.42 ± 0.29	+31.92 ± 2.20	+0.60 ± 0.29	+57.20 ± 1.31	+1.16 ± 0.34
EPDM 40	+22.21 ± 1.23	+0.56 ± 0.21	+33.34 ± 0.77	+1.47 ± 0.15	+60.17 ± 3.22	+1.95 ± 0.36
EPDM 50	+60.21 ± 3.17	+2.58 ± 0.37	+121.93 ± 5.42	+5.12 ± 0.25	+306.31 ± 5.86	+7.78 ± 0.36
Acetic acid concentration 20%						
EPDM 0	+11.65 ± 0.38	−0.67 ± 0.38	+15.02 ± 0.48	−1.33 ± 0.34	+11.94 ± 0.58	−2.39 ± 0.26
EPDM 10	+15.27 ± 1.04	−0.47 ± 0.01	+21.49 ± 0.68	−0.66 ± 0.01	+29.05 ± 2.64	−0.30 ± 0.12
EPDM 20	+19.29 ± 1.58	−0.59 ± 0.17	+25.12 ± 1.35	−0.63 ± 0.25	+29.75 ± 0.75	−1.60 ± 0.43
EPDM 30	+20.53 ± 0.60	+0.51 ± 0.36	+33.05 ± 0.86	+0.65 ± 0.23	+60.09 ± 1.27	+1.30 ± 0.21
EPDM 40	+22.52 ± 1.16	+0.73 ± 0.46	+37.01 ± 1.42	+1.56 ± 0.23	+66.36 ± 1.51	+2.01 ± 0.62
EPDM 50	+66.92 ± 4.31	+3.38 ± 0.36	+128.94 ± 6.07	+6.54 ± 0.27	+338.19 ± 12.37	+8.16 ± 0.81
Acetic acid concentration 30%						
EPDM 0	+10.58 ± 0.98	−0.45 ± 0.22	+12.94 ± 0.98	−1.51 ± 0.42	+12.27 ± 1.65	−2.80 ± 0.66
EPDM 10	+15.13 ± 1.25	−0.48 ± 0.02	+20.95 ± 1.03	−0.65 ± 0.43	+28.49 ± 0.85	−0.62 ± 0.31
EPDM 20	+17.78 ± 0.65	−0.60 ± 0.14	+25.05 ± 1.03	−1.10 ± 0.27	+25.12 ± 2.41	−2.38 ± 0.58
EPDM 30	+21.45 ± 0.35	+0.81 ± 0.22	+37.26 ± 1.07	+1.16 ± 0.28	+64.97 ± 1.50	−0.47 ± 0.48
EPDM 40	+24.45 ± 1.19	+1.54 ± 0.16	+40.29 ± 1.53	+1.62 ± 0.12	+74.23 ± 1.39	+2.33 ± 0.43
EPDM 50	+70.93 ± 4.31	+4.92 ± 0.51	+151.05 ± 8.51	+7.68 ± 0.41	+398.90 ± 24.25	+8.39 ± 0.28

**Table 4:** Cross-link densities of EPDM-based composites after immersion in acetic acid solutions (q × 10^−4^ mol/cm^3^) and the percentage changes (%) relative to untreated samples.

Sample Code	3 Days		7 Days		14 Days	
	Cross-Link Density	Cross-Link Density Modification	Cross-Link Density	Cross-Link Density Modification	Cross-Link Density	Cross-Link Density Modification
Acetic acid concentration 10%						
EPDM 0	0.1981 ± 0.0118 ^fA^	+14.57	0.2099 ± 0.0089 ^eA^	+21.40	0.1423 ± 0.0121 ^eAB^	−17.70
EPDM 10	2.1041 ± 0.0110 ^eA^	+30.14	2.1410 ± 0.0351 ^dA^	+32.42	1.5915 ± 0.0232 ^dAB^	−1.56
EPDM 20	3.8544 ± 0.1753 ^bA^	+4.46	3.5258 ± 0.0731 ^aA^	−4.45	3.3327 ± 0.1850 ^aA^	−9.68
EPDM 30	3.4299 ± 0.0564 ^cA^	+18.48	3.2086 ± 0.1413 ^bA^	+10.84	2.9252 ± 0.0826 ^bA^	+1.05
EPDM 40	2.7109 ± 0.1184 ^dA^	+22.39	2.4496 ± 0.0368 ^cA^	+10.60	2.3462 ± 0.0489 ^cA^	+5.93
EPDM 50	4.1766 ± 0.0597 ^aA^	+90.07	3.6865 ± 0.0969 ^aA^	+67.77	3.0833 ± 0.1045 ^abA^	+40.32
*F* value	740.57522		803.01025		471.19999	
*p* value	<0.0001		<0.0001		<0.0001	
Acetic acid concentration 20%						
EPDM 0	0.1915 ± 0.0052 ^cA^	+10.76	0.1782 ± 0.0117 ^fB^	+3.07	0.1405 ± 0.0040 ^dB^	−18.74
EPDM 10	2.1456 ± 0.0311 ^bA^	+32.71	1.7813 ± 0.0261 ^eB^	+10.17	1.4699 ± 0.0173 ^cB^	−9.09
EPDM 20	3.8243 ± 0.1425 ^aA^	+3.64	3.3944 ± 0.1662 ^bA^	−8.01	3.2202 ± 0.0973 ^aA^	−12.73
EPDM 30	3.4206 ± 0.0851 ^abA^	+18.16	2.9947 ± 0.0844 ^cA^	+3.45	2.8531 ± 0.0783 ^aA^	−1.44
EPDM 40	2.6775 ± 0.1141 ^abA^	+20.89	2.3418 ± 0.0345 ^dB^	+5.73	2.2555 ± 0.1510 ^bA^	+1.83
EPDM 50	4.0036 ± 1.3812 ^aA^	+82.20	3.7101 ± 0.1539 ^aA^	+68.84	2.9614 ± 0.2692 ^aA^	+34.77
*F* value	18.62194		498.89353		220.45892	
*p* value	<0.0001		<0.0001		<0.0001	
Acetic acid concentration 30%						
EPDM 0	0.1903 ± 0.0048 ^eA^	+10.06	0.1798 ± 0.0081 ^fB^	+3.99	0.1681 ± 0.0127 ^eA^	−2.78
EPDM 10	1.9235 ± 0.0214 ^dB^	+18.97	1.7099 ± 0.0315 ^eB^	+5.76	1.6414 ± 0.1148 ^dA^	+1.52
EPDM 20	3.8045 ± 0.2803 ^aA^	+3.10	3.4708 ± 0.0775 ^bA^	−5.94	3.2546 ± 0.1391 ^aA^	−11.80
EPDM 30	3.2787 ± 0.12006 ^bA^	+13.26	2.9609 ± 0.0654 ^cA^	+2.28	2.7508 ± 0.0409 ^bA^	−4.98
EPDM 40	2.6716 ± 0.1161 ^cA^	+20.62	2.2151 ± 0.0538 ^dC^	+0.01	2.0789 ± 0.1853 ^cA^	−6.14
EPDM 50	4.0434 ± 0.1647 ^aA^	+84.01	3.7786 ± 0.1814 ^aA^	+71.96	2.8274 ± 0.2206 ^bA^	+28.67
*F* value	275.29075		671.96274		190.98597	
*p* value	<0.0001		<0.0001		<0.0001	

The values represent the averages of three independent samples. Values within each column marked with different lowercase/uppercase superscripts are significantly different (*p* ≤ 0.05). Lowercase letters are associated with comparisons made vertically (on the column), and uppercase letters, horizontally (on the line). Statistical analysis was performed using one-way ANOVA followed by Fisher’s Least Significant Difference (LSD) post hoc test. A significance level of *p* < 0.05 indicates significant differences between sample means; *p* > 0.05 indicates no significant difference. For the ANOVA test: number of groups (k) = 6, number of samples (n) = 18, degrees of freedom for the numerator (df_1_) = 5, and for the denominator (df_2_) = 12. The F-critical value is 3.1059.

**Table 5:** Young’s modulus (*E*, MPa) of composites and its percentage variation (%) after immersion in acetic acid solutions.

Sample Code	3 Days		7 Days		14 Days	
	Young’s Modulus	Young’s Modulus Variation	Young’s Modulus	Young’s Modulus Variation	Young’s Modulus	Young’s Modulus Variation
Acetic acid concentration 10%						
EPDM 0	3.85 ± 0.75	−23.46	3.43 ± 0.89	−31.81	3.79 ± 0.59	−24.73
EPDM 10	4.22 ± 0.33	+2.48	4.10 ± 0.68	−0.34	3.08 ± 0.82	−25.25
EPDM 20	6.25 ± 1.30	−7.00	6.45 ± 0.51	−3.96	6.82 ± 1.64	+1.55
EPDM 30	6.63 ± 1.06	−4.30	6.60 ± 0.87	−4.71	6.72 ± 0.46	−3.02
EPDM 40	7.34 ± 0.94	−9.74	5.81 ± 1.03	−28.54	6.11 ± 0.92	−24.90
EPDM 50	8.98 ± 1.64	−1.47	6.70 ± 0.72	−26.52	4.71 ± 0.47	−48.31
Acetic acid concentration 20%						
EPDM 0	4.20 ± 0.53	−16.46	3.78 ± 0.48	−24.93	3.79 ± 0.46	−20.32
EPDM 10	3.32 ± 0.22	−19.28	3.55 ± 0.51	−13.89	3.83 ± 0.36	−13.61
EPDM 20	5.99 ± 0.64	−10.87	5.91 ± 0.36	−11.97	6.47 ± 1.09	+5.42
EPDM 30	6.52 ± 1.05	−5.78	5.81 ± 0.77	−16.15	6.10 ± 0.41	−0.95
EPDM 40	6.41 ± 0.49	−21.13	6.09 ± 0.95	−25.07	6.47 ± 0.93	−19.07
EPDM 50	7.69 ± 1.22	−15.64	6.13 ± 0.71	−32.80	3.99 ± 0.82	−39.27
Acetic acid concentration 30%						
EPDM 0	4.21 ± 0.73	−16.34	4.01 ± 0.31	−20.32	3.20 ± 0.25	−36.46
EPDM 10	4.23 ± 0.90	+2.72	3.56 ± 0.30	−13.61	4.26 ± 0.56	+3.55
EPDM 20	6.39 ± 1.00	−4.91	7.08 ± 1.20	+5.42	6.58 ± 0.61	−2.00
EPDM 30	6.28 ± 0.73	−9.33	6.86 ± 0.37	−0.95	5.92 ± 1.30	−14.44
EPDM 40	6.17 ± 0.76	−24.08	6.58 ± 1.03	−19.07	6.42 ± 0.27	−21.08
EPDM 50	7.84 ± 1.47	−14.02	5.54 ± 1.49	−39.27	3.74 ± 0.73	−59.02

**Table 6:** Tensile strength of composites, *σ* (MPa), and its percentage variation (%) after immersion in acetic acid solutions.

Sample Code	3 Days		7 Days		14 Days	
	Tensile Strength	Tensile Strength Variation	Tensile Strength	Tensile Strength Variation	Tensile Strength	Tensile Strength Variation
Acetic acid concentration 10%						
EPDM 0	10.21 ± 0.93	+63.53	6.59 ± 1.07	+5.57	6.98 ± 0.72	+11.82
EPDM 10	1.04 ± 0.06	−70.73	1.00 ± 0.05	−71.75	1.02 ± 0.04	−71.11
EPDM 20	2.79 ± 0.40	−9.36	2.57 ± 0.13	−16.50	2.81 ± 0.32	−8.64
EPDM 30	1.62 ± 0.16	+0.75	1.55 ± 0.11	−3.62	1.90 ± 0.07	+18.45
EPDM 40	1.21 ± 0.06	−19.31	1.17 ± 0.07	−21.97	1.17 ± 0.05	−22.37
EPDM 50	1.02 ± 0.07	+0.63	0.91 ± 0.06	−10.22	0.72 ± 0.05	−28.92
Acetic acid concentration 20%						
EPDM 0	9.42 ± 0.59	+50.88	6.52 ± 0.70	+4.42	7.63 ± 0.43	+22.13
EPDM 10	1.07 ± 0.11	−69.79	0.89 ± 0.07	−74.87	1.03 ± 0.13	−70.83
EPDM 20	3.31 ± 0.27	+7.54	2.38 ± 0.29	−22.68	2.96 ± 0.52	−3.83
EPDM 30	1.86 ± 0.14	+15.84	1.41 ± 0.13	−11.97	1.97 ± 0.28	+22.57
EPDM 40	1.17 ± 0.02	−22.10	1.12 ± 0.05	−25.57	1.15 ± 0.03	−23.30
EPDM 50	0.93 ± 0.07	−8.33	0.94 ± 0.03	−7.21	0.89 ± 0.14	−12.34
Acetic acid concentration 30%						
EPDM 0	9.28 ± 0.13	+48.58	6.56 ± 0.85	5.03	6.09 ± 0.76	−2.43
EPDM 10	1.04 ± 0.06	−70.56	1.13 ± 0.14	−67.99	1.15 ± 0.16	−67.46
EPDM 20	2.80 ± 0.48	−9.03	2.94 ± 0.25	−4.65	3.10 ± 0.29	+0.80
EPDM 30	1.63 ± 0.25	+1.37	1.74 ± 0.25	+8.35	1.76 ± 0.15	+9.85
EPDM 40	1.14 ± 0.06	−24.37	1.18 ± 0.08	−21.70	1.12 ± 0.08	−25.17
EPDM 50	0.96 ± 0.12	−5.67	0.93 ± 0.08	−8.72	0.82 ± 0.05	−19.37

**Table 7:** Specific elongation of composites, *ε* (%), and its percentage variation (%) after immersion in acetic acid solutions.

Sample Code	3 Days		7 Days		14 Days	
	Specific Elongation	Specific Elongation Variation	Specific Elongation	Specific Elongation Variation	Specific Elongation	Specific Elongation Variation
Acetic acid concentration 10%						
EPDM 0	942 ± 29	−3.48	846 ± 47	−13.32	854 ± 61	−12.50
EPDM 10	445 ± 54	−23.80	412 ± 64	−29.45	468 ± 52	−19.86
EPDM 20	324 ± 55	−1.82	304 ± 18	−7.88	336 ± 21	+1.82
EPDM 30	230 ± 52	−6.50	224 ± 24	−8.94	308 ± 10	+25.00
EPDM 40	144 ± 25	−51.68	144 ± 37	−51.81	162 ± 47	−45.64
EPDM 50	61 ± 27	−43.50	96 ± 19	−11.84	110 ± 10	+1.23
Acetic acid concentration 20%						
EPDM 0	950 ± 19	−2.66	824 ± 51	−15.57	908 ± 32	−6.97
EPDM 10	482 ± 50	−17.47	423 ± 33	−27.65	440 ± 47	−24.66
EPDM 20	400 ± 22	+21.21	293 ± 51	−11.36	348 ± 56	+5.30
EPDM 30	280 ± 32	+13.82	178 ± 38	−27.64	310 ± 48	+26.02
EPDM 40	184 ± 21	−38.26	150 ± 36	−49.66	160 ± 28	−46.31
EPDM 50	61 ± 22	−43.68	104 ± 11	−4.66	134 ± 53	+23.31
Acetic acid concentration 30%						
EPDM 0	1006 ± 53	+3.07	804 ± 59	−17.62	626 ± 64	−35.86
EPDM 10	487 ± 67	−16.67	510 ± 62	−12.67	415 ± 38	−28.94
EPDM 20	353 ± 43	+6.82	330 ± 35	+0.00	328 ± 51	−0.61
EPDM 30	258 ± 65	+4.88	272 ± 53	+10.57	270 ± 26	+9.76
EPDM 40	146 ± 51	−51.14	175 ± 60	−41.14	158 ± 25	−46.98
EPDM 50	61 ± 22	−44.33	117 ± 39	+7.44	106 ± 8	−2.22

**Table 8:** FTIR band assignments associated with functional groups present in wood sawdust and EPDM components as observed in the FTIR-ATR spectra of EPDM 30 and EPDM 20 composites.

Wavelength Band (cm^−1^)	Assignment
2953, 2918, 2849	-C-H stretching vibration (2800–3000 cm^−1^) [86,87].
1539	Aromatic skeletal vibrations caused by lignin and C=O stretch in non-conjugated ketones, carbonyls, and in ester groups [88].
1461	-CH_2_ bending and rocking vibrations [86,87].
1398	Stretching and bending vibrations of -CH_2_ bonds, associated with the amount of the crystalline structure of the cellulose [89,90].
1377	-CH_3_ bending vibration [86,87].
1098	-C-O stretching vibrations [91]
953, 890	-O-O stretching vibration [91]
803	-C-O bonds in cellulose [89,90]
722	-CH_2_ bending and rocking vibrations [86,87].

**Table 9:** Summary of Thermogravimetric Analysis (TGA) results.

Sample Code and Treatment Conditions	W_1_ (%)	T_1_ (°C)	W_2_ (%)	T_2_ (°C)	W_3_ (%)	T_3_ (°C)	T_DSC_ (°C)
EPDM 0 EPDM 10 EPDM 20 EPDM 30 EPDM 40 EPDM 50	- - - 6.6 5.0 6.9	- - - 306 305 299	- - 11.4 13.3 11.1 14.6	- - 366 360 354 361	70.5 72.6 64.5 69.9 67.1 54.1	475 476 475 474 474 471	472 472 473 468 470 471
EPDM 20: 10% AA, 3 days EPDM 20: 10% AA, 14 days EPDM 20: 30% AA, 3 days EPDM 20: 30% AA, 14 days	- - - -	- - - -	11.8 14.9 14.5 14.3	361 358 359 365	63.8 62.2 61.5 62.7	476 503 477 477	472 472 472 474
EPDM 30: 10% AA, 3 days EPDM 30: 10% AA, 14 days EPDM 30: 30% AA, 3 days EPDM 30: 30% AA, 14 days	- - - -	- - - -	15.6 13.9 19.4 16.1	359 358 359 360	63.9 65.8 59.1 63.8	477 473 473 474	471 473 471 470

AA—acetic acrylic; T_1/2/3_—temperatures corresponding to 1/2/3 mass losses; W_1/2/3_—mass loss per each stage 1/2/3; T_DSC_—temperatures from DSC.

## Data Availability

All the results are presented in the manuscript.
